# Effective Expression of the *Serratia marcescens* Phospholipase A1 Gene in *Escherichia coli* BL21(DE3), Enzyme Characterization, and Crude Rapeseed Oil Degumming via a Free Enzyme Approach

**DOI:** 10.3389/fbioe.2019.00272

**Published:** 2019-10-17

**Authors:** Peizhou Yang, Yun Wu, Suwei Jiang, Zhi Zheng, Zhigang Hou, Dongdong Mu, Wei Xiao, Shaotong Jiang, Yung-Hun Yang

**Affiliations:** ^1^Anhui Key Laboratory of Intensive Processing of Agricultural Products, College of Food and Biological Engineering, Hefei University of Technology, Hefei, China; ^2^Department of Biological, Food and Environment Engineering, Hefei University, Hefei, China; ^3^Department of Biological Engineering, College of Engineering, Konkuk University, Seoul, South Korea

**Keywords:** *Serratia marcescens*, phospholipase A, enzymatic degumming, rapeseed oil, enzyme characterization, Lecitase Ultra

## Abstract

Crude oil degumming by phospholipid removal is crucial to guarantee oil quality. Phospholipase degumming could produce green vegetable oil by reducing energy consumption and protecting the environment. To develop a novel phospholipase for oil degumming, we cloned the *Serratia marcescens* outer membrane phospholipase A gene (*OM-PLA1*) and expressed its 33 KDa protein in engineered *Escherichia coli* BL21(DE3). OM-PLA1 activity reached 18.9 U mL^−1^ with the induction of 0.6 mM isopropyl β-D-1-thiogalactopyranoside for 4 h. The optimum temperature and pH were 50°C and 7.5, respectively. Mg^2+^, Ca^2+^, Co^2+^, and Mn^2+^ at 0.1 mM L^−1^ significantly increased OM-PLA1 activity. The kinetic equations of OM-PLA1 and Lecitase Ultra were *y* = 13.7*x*+0.74 (K_m_ = 18.53 mM, V_max_ = 1.35 mM min^−1^) and *y* = 24.42*x*+0.58 (K_m_ = 42.1 mM, V_max_ = 1.72 mM min^−1^), respectively. The phosphorus content decreased from 22.6 to 9.3 mg kg^−1^ with the addition of 15 units of free recombinant OM-PLA1 into 150 g of crude rapeseed oil. OM-PLA1 has the close degumming efficiency with Lecitase Ultra. The *S. marcescens* outer membrane phospholipase gene (OM-PLA1) possessed higher substrate affinity and catalytic efficiency than Lecitase Ultra. This study provides an alternative approach to achieve crude vegetable oil degumming with enzymatic technology.

## Introduction

Crude vegetable oil mainly comprises trace elements, free fatty acids, triglycerides, sterols, phospholipids, heavy metals, and other minor impurities (More and Gogate, 2018). Phospholipids are negative components in vegetable oils during oil deodorization and steam distillation (Cmolík and Pokorný, 1995; Bora, 2013). When heated, phospholipids produce foam, smoke, and brown substances. These substances affect the senses of fried foods and are dangerous when used. In addition, the oxidized products of phospholipids affect the performance and nutrition of edible oil and produce substances that are harmful to human health (Navab et al., 2004; Que et al., 2018). As an important step in refining vegetable oil, degumming can remove the phospholipids dissolved in crude oils (Dijkstra, 2010), maintain the quality and stability of oil for long-term storage (Sampaio et al., 2015), increase the refining efficiency of edible oil, and enhance the quality of products (Sampaio et al., 2019; Yang et al., 2019). Chemical degumming can remove non-hydrable phospholipids but simultaneously cause saponin formation, considerable oil loss, and environmental pollution (Dijkstra, 2017). Phospholipases A1, A2, or B can wet-fine the crude vegetable oils to remove mucilage, and then the aqueous phase is removed from the oil (Aalrust et al., 1992). Therefore, phospholipase degumming is an alternative approach that is more effective and environment friendly than chemical degumming (Jiang et al., 2014a).

Various phospholipases have been used in vegetable oil degumming (Huang et al., 2014; Cerminati et al., 2017; Bornscheuer, 2018). Phospholipase A1 hydrolyzes phosphatidyl groups to produce lyso- phosphatidylcholine and free fatty acids in many cells from various organisms (Yu et al., 2013). However, a limited number of phospholipase A1 genes have been purified so far. These genes include serine phospholipid-specific phospholipase A from rat platelets (Sato et al., 1997), phospholipase A1 from wasp venom (King et al., 1984) and *Manduca sexta* (Arrese et al., 2006), phospholipase from *Fusarium oxysporum* (Clausen et al., 1998), and phosphatidic acid-selective phospholipase from human testis (Hiramatsu et al., 2003). In addition, various phospholipase A1 genes are expressed in animal pancreas (Weide et al., 2006; Harris and Scott, 2013), *Bacillus cereus* (Fu et al., 2008), *Saccharomyces cerevisiae* and *Aspergillus oryzae* (Shiba et al., 2001), yeast strains (Noiriel et al., 2004), and *Arabidopsis* (Seo et al., 2008).

**Figure 1:**
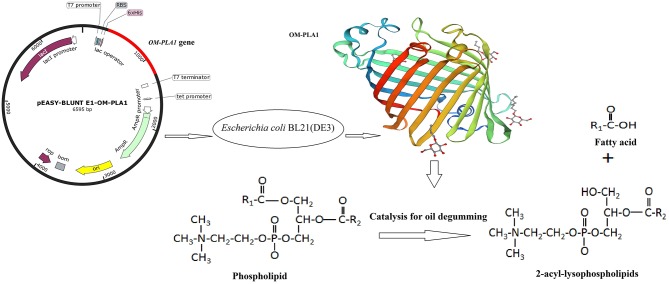
Scheme of this study. The expression vector *Escherichia coli* BL21(DE3) was integrated by OM-PLA1 expression recombinant OM-PLA1. The recombinant enzyme can catalyze phospholipids to produce 2-acyl-lysophospholipids and fatty acids for crude oil degumming.

Rapeseed oil is a typical vegetable oil containing high phospholipid content before refining. In crude rapeseed oil, the total phosphatide and phosphorus contents can reach 972 and 1,176 mg kg^−1^, respectively (Dijkstra, 2016). The food industry requires a phosphorus content not exceeding 10 mg kg^−1^ (Sheelu et al., 2008; Yu et al., 2012; Sampaio et al., 2015). The low activity of phospholipase A1 in natural sources cannot satisfy the demands for industrial production. The construction of an engineered strain is an effective approach to overexpress phospholipase genes (Shiba et al., 2001; Noiriel et al., 2004; Seo et al., 2008). In the current study, the *Serratia marcescens* OM-PLA1 gene was transformed into *Escherichia coli* BL21(DE3) for heterologous expression. The enzymatic characteristics of OM-PLA1 and free enzymatic degumming of rapeseed oil were also investigated (Figure 1). In addition, the enzymatic characteristics and degumming performance of OM-PLA1 and commercialized phospholipase Lecitase Ultra were compared. This study could provide an enzyme source for the application of *S. marcescens* OM-PLA1 in the enzymatic degumming of crude rapeseed oils.

## Materials and Methods

### Materials, Reagents, and Devices

The *Serratia marcescens* outer membrane phospholipase gene (*OM-PLA1*) was cloned and preserved in the College of Food and Biological Engineering, Hefei University of Technology. The biological and chemical reagents, pEASY-T1 cloning kit, pEASY-Blunt E1 expression kit, *Escherichia coli* DH *5*α, and *E. coli* BL21(DE3) were purchased from Transgen Biotech. The gel imaging system and the PCR amplification, SDS-PAGE, and electrophoresis devices were obtained from Bio-RAD. Primer synthesis and gene sequencing were performed by Sangon Corporation.

### Expression Vector and Engineered *E. coli* BL21(DE3)

The upstream primer 5′-TATGCGCATTTTGTCAGGGA-3′ and downstream primer 5′-GATTACATAATATCGTTCAGC-3′ based on the Genbank sequence HG326223.1 were used to amplify the *S. marcescens* OM-PLA1 gene. A pEASY-T1 cloning vector with the OM-PLA1 gene was transformed into *E. coli* DH*5*α for sequencing identification. The confirmed *S. marcescens* OM-PLA1 gene was inserted into a pEASY-Blunt E1 expression vector. T7 promoter primer and T7 terminator primer were used to amplify the insertion sequence for sequencing. The vector embracing *OM-PLA1* with correct insertion location was identified. The upstream sequence of *OM-PLA1* is close to the T7 promoter. The identified recombinant expression vector was transformed into *E. coli* BL21(DE3) for heterologous expression.

### OM-PLA1 Recombinant Expression

The expression of OM-PLA1 in engineered *E. coli* BL21(DE3) was induced by adding IPTG in an expression broth. The OM-PLA1 engineered *E. coli* BL21(DE3) was inoculated into a 50 mL Erlenmeyer flask loaded with 5 mL of LB liquid medium containing 100 μg of mL ampicillin at 37°C and 250 rpm shaking speed. When the cell concentration was 0.5 OD600, 100 mM IPTG was sucked out and injected into the Erlenmeyer flask. The condensed IPTG was diluted to 0–1.2 mM for the induction of recombinant OM-PLA1 at 37°C and 250 rpm shaking speed. The broth was centrifuged at 10,000 rpm for 5 min, and then the upper broth was taken for the activity detection of OM-PLA1. The effects of 0–8 h of induction time on OM-PLA1 expression were also investigated.

### Purification and SDS-PAGE Analysis

The pEASY-Blunt E1 expression vector contained a 6× His protein purification label at its N terminal. The label helped purify the recombinant protein conveniently. The recombinant OM-PLA1 was purified using ProteinIso Ni-IDA Resin (Transgen Biotech company) in accordance with the product protocol. IDA can chelate Ni^2+^ firmly through three sites and reduce the leakage of Ni^2+^ to protein samples during purification. ProteinIso Ni-IDA Resin has a specific adsorption capacity for the labeled proteins. The labeled proteins were bound to NI-IDA purification medium, whereas the unbounded proteins were washed down. The detailed processing steps are as follows: (1) ProteinIso Ni-IDA Resin was re-suspended with an equilibrium liquid containing 50 mM NaH_2_PO_4_, 300 mM NaCl, 10 mM imidazole, and 10 mM Tris base adjusted to pH 8; (2) the supernatant of the fermentation broth was obtained by centrifugation at 10,000 rpm for 10 min. The supernatant was further filtered through a 0.45 μm aperture. The filtered sample was prepared by diluting the supernatant 10 times; (3) after sampling, the column was washed with 10-fold equilibrium liquid; (4) the target protein was eluted by adding equilibrium liquid adjusted to pH 5. The protein profile of *S. marcescens* OM-PLA1 eluted from the resin was analyzed via SDS-PAGE (Yang et al., 2016).

### Enzymatic Characteristic Analysis

The kinetic equations of OM-PLA1 and Lecitase Ultra were drawn using 1/[S] and 1/V_0_ as abscissa and ordinate, respectively. K_m_ (mM) and V_max_ (mM min^−1^) were calculated using Lineweaver–Burk method (Chethankumar and Srinivas, 2008; Dijkstra, 2010). The conditions of pH 7.5 and 50°C were, respectively, maintained to investigate the effect of temperature and pH on enzyme activity. The enzymatic characteristics of OM-PLA1 in terms of optimum temperature, optimum pH, temperature stability, pH tolerance, and kinetic parameters were determined by measuring enzymatic activities under different conditions (An et al., 2017). The Arrhenius plot method was used to analyze the effect of temperature on the stabilization rate of OM-PLA1 by drawing the relationship between the initial rate of enzymatic hydrolysis and reaction temperature (Fan et al., 2000).

The effects of certain concentrations of factors on the enzyme activities were investigated. The activities were measured after the addition of 0.1 mM of Ca^2+^, Mg^2+^, Co^2+^, and Mn^2+^ (Ben et al., 2013) and 1 mM of K^+^, Na^+^, Cu^2+^, Zn^2+^, Al^3+^, EGTA (Kornspan and Rottem, 2012), EDTA, and SDS (Hsu et al., 2014) in Triton X-100 solution. In addition, the control was used with Triton X-100 solution as buffer. The relative activities of OM-PLA1 (%) were calculated by dividing the enzyme activities of different factors with the control multiplied by 100% (Jiang et al., 2015). A concentration of 1 mM Mg^2+^ affecting the secondary structure of OM-PLA1 was investigated by circular dichroism (CD) spectroscopy (Koubaa et al., 2019; Yi et al., 2019). The CD spectra of OM-PLA1 were obtained by Chirascan qCD (Applied Photophysics Ltd., UK). Approximately 0.5 mg/mL OM-PLA1 was measured in a 0.5 mm path length cuvette. The data were recorded from 190 to 260 nm with a time-per-point of 1 s and an interval of 1 nm (Koubaa et al., 2019). CDPro software was used to analyze the data (Yi et al., 2019).

### Crude Oil Degumming via the Free OM-PLA1 Approach

Crude oil degumming was investigated by measuring phosphorus content (Jiang et al., 2014b). Approximately 0.24 mL of 45% citric acid (w/v) was added into 150 g of crude oils. The mixture was blended under a shaking speed of 1,000 rpm at 80°C for 20 min. The mixture solution was added with 0.5 mL of free OM-PLA1 and 3 mL of distilled water for enzymatic degumming. The reaction of enzymatic degumming was terminated at 90°C for 10 min. The effect of degumming was represented on the basis of residual phosphorus content. Phosphorus content was measured in accordance with the AOCS Official Method Ca 12–55 (Reapproved in 2009) (Chen et al., 2014).

### Enzyme Activity Determination

OM-PLA1 and Lecitase Ultra activities were measured with soybean lecithin as the catalytic substrate (Sheelu et al., 2008). A mixture of 180 mL of 0.02 M citric acid and 20 mL of 0.01 M disodium hydrogen phosphate was prepared as Triton X-100 solution. The mixture used for catalyzing the substrate was prepared by adding 10 g of soybean lecithin into 200 mL of Triton X-100 solution. A mixture of 98 mL of catalysis substrate and 2 mL of free enzyme was bathed at 50°C for 15 min at a shaking speed of 180 rpm. Catalysis was terminated by adding 15 mL of 95% ethanol (w/v). The reaction solution was adjusted to pH 10 by adding 25 mM NaOH. The volume of consumed NaOH was used to measure OM-PLA1 activity.

### Data Analysis

Three replicates were used in each data point in this study. All results in the figures are presented as means ± standard deviation by using the Origin software.

## Results

### Recombinant Expression of OM-PLA1 in Engineered *E. coli* BL21(DE3)

**Figure 2:**
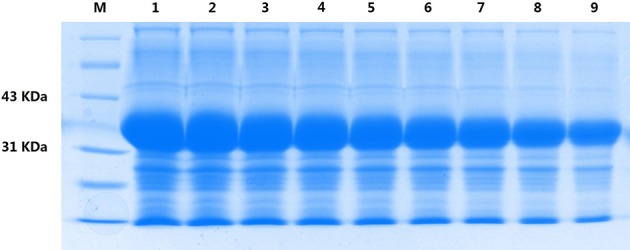
Profile of recombinant OM-PLA1 of engineered *E. coli* BL21(DE3) based on SDS-PAGE analysis with 0.6 mM IPTG induction under different treatment times. Lane M, Protein marker; lanes 1–9 indicate treatment times of 4.5–0.5 h every 0.5 h.

The *S. marcescens* OM-PLA1 gene of 879 bp in size was amplified. The amplification product was inserted into the pEASY-Blunt E1 plasmid. After sequencing confirmation, the correct expression vector containing the OM-PLA1 gene was transformed into *E. coli* BL21(DE3). The protein profiles determined via SDS-PAGE showed that the size of OM-PLA1 expressed by engineered *E. coli* BL21(DE3) was appropriately 33 KDa. With the addition of 0.6 mM IPTG, SDS-PAGE indicated that the expression level of the recombinant OMP-LA1 positively increased when the treatment time was 0.5–4.5 h (Figure 2).

### Induction of IPTG on OM-PLA1 Expression

**Figure 3:**
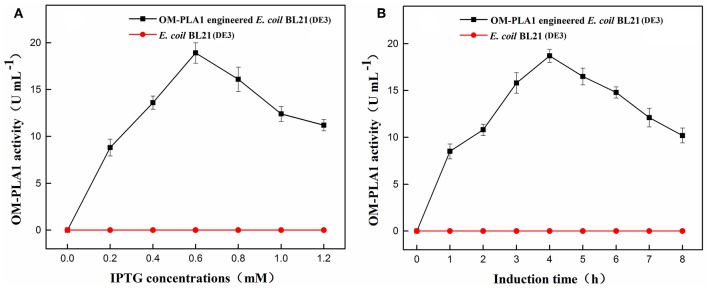
Effect of IPTG concentration **(A)** and induction time **(B)** on outer membrane phospholipase A1 activity. U of phospholipase activity was defined to hydrolyze phospholipids for 1 min to produce 1 μmol of free fatty acids under certain conditions.

The effects of IPTG concentration and induction time on OM-PLA1 activity were investigated. The concentration of 0.6 mM IPTG resulted in the highest expression efficiency of OM-PLA1 among the set concentrations of 0–1.2 mM. After 0.6 mM IPTG treatment for 4 h, the OM-PLA1 activity of engineered *E. coli* BL21(DE3) reached 18.9 U mL^−1^, which was the highest among the set concentrations of IPTG. Almost no OM-PLA1 activity in the broth from wild-type *E. coli* BL21(DE3) was observed in the control (Figure 3A). After 4 h of induction with the addition of 0.6 mM IPTG, the activity of OM-PLA1 expressed by the engineered *E. coli* BL21(DE3) reached 18.7 U mL^−1^, which was the highest concentration achieved among the set treatment times (Figure 3B).

### Treatment Time Affecting the Residual Activity of OM-PLA

**Figure 4:**
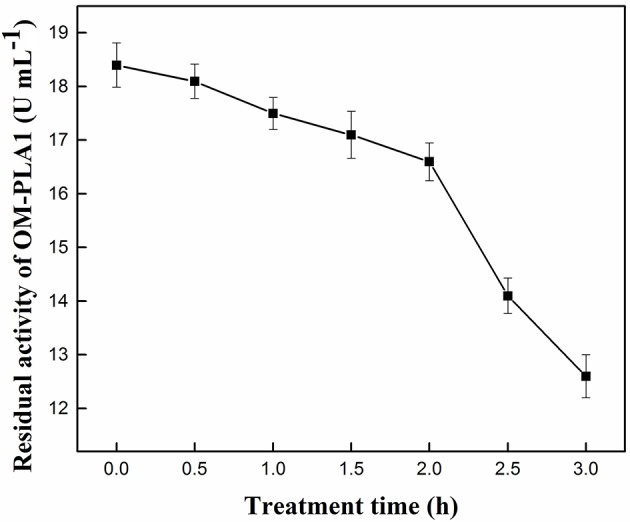
Effect of treatment time on the residual activity of OM-PLA.

Under the conditions of 50°C and pH 7.5, the effect of treatment time on the residual activity of OM-PLA1 was investigated (Figure 4). The decreasing range of enzyme activity was less under the 0–2 h treatment than under the 2–3 h activity. OM-PLA1 activity remarkably decreased after more than 2 h. Treatment time of 2 h was selected to investigate enzyme stability under different temperatures and pH levels. The residual enzyme activity of 89.66% indicated that OM-PLA1 still possessed good enzymatic stability after a treatment of 2 h.

### Kinetic Parameter Determination

**Figure 5:**
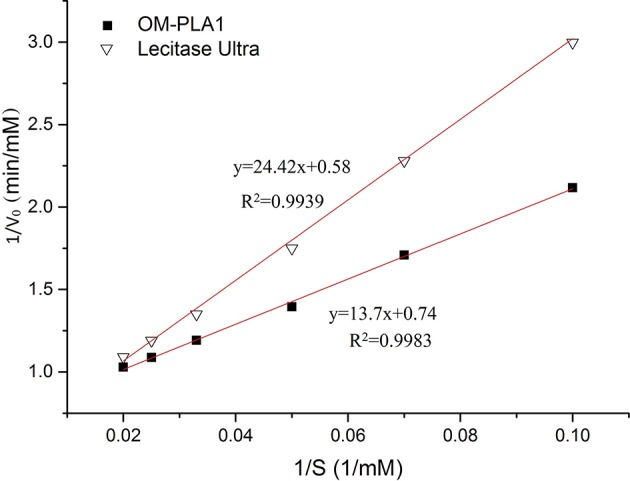
Kinetic equations of OM-PLA1 and Lecitase Ultra based on Lineweaver–Burk equation.

The kinetic equations of OM-PLA1 and Lecitase Ultra were drawn based on Lineweaver–Burk equation under the same conditions (Figure 5). The kinetic equation of OM-PLA1 was *y* = 13.7*x*+0.74 (*R*^2^ = 0.9983, K_m_ = 18.53 mM, and V_max_ = 1.35 mM min^−1^). The kinetic equation of Lecitase Ultra was *y* = 24.42*x*+0.58 (*R*^2^ = 0.9939, K_m_ = 42.1 mM, V_max_ = 1.72 mM min^−1^). By contrast, OM-PLA1 possessed lower enzyme activity and higher substrate affinity than Lecitase Ultra.

### Temperature and pH Affecting OMP-PLA1 Activities

**Figure 6:**
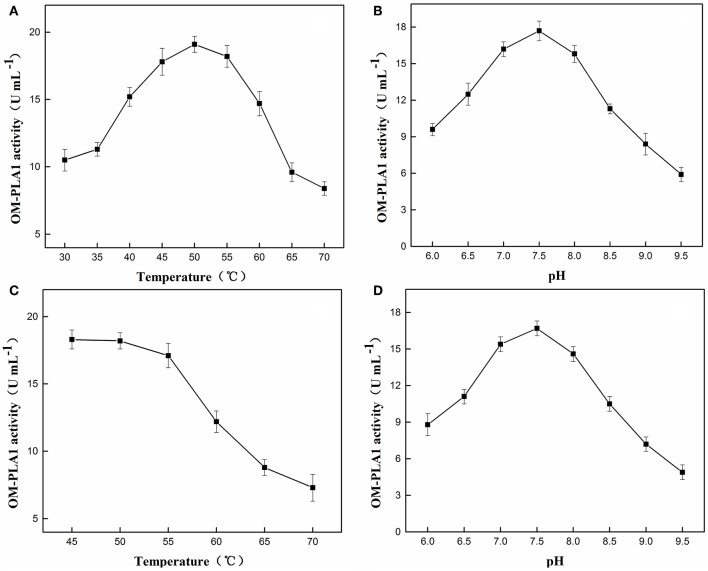
Effect and enzyme stability of temperatures and pH values on OM-PLA1. Effect of different temperatures **(A)** and pH values **(B)** on OM-PLA1 activity; enzyme stability of OM-PLA1 under different temperatures **(C)**, and pH values **(D)** after treatment for 2 h.

The enzymatic characteristics of OM-PLA1 were analyzed by investigating the optimum temperature and pH and the thermal and pH tolerance. The highest enzymatic activities at 50°C and pH 7.5 were 19.1 U (Figure 6A) and 17.7 U mL^−1^ (Figure 6B), respectively. After treatment for 2 h, the good stability of OM-PLA1 activity was maintained at 50°C (Figure 6C) and pH 7.5 (Figure 6D), and the highest enzymatic activities under such conditions were 18.2 and 16.7 U mL^−1^, respectively.

**Figure 7:**
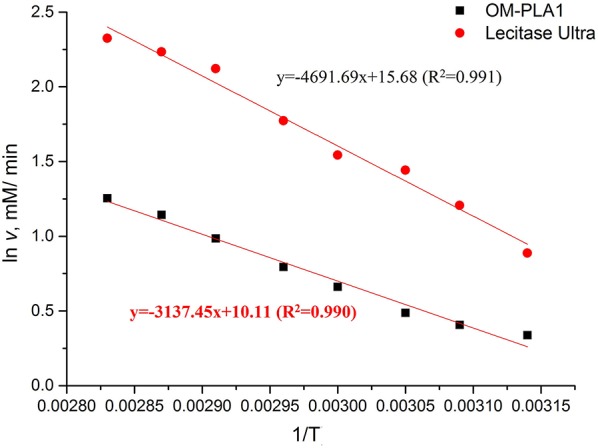
Arrhenius plot method analyzing the relationship between the initial rate of enzymatic hydrolysis and reaction temperature.

The Arrhenius plot method was used to investigate the relationship between initial hydrolysis speed and reaction temperature (Figure 7). The Arrhenius plot equations of OM-PLA1 and Lecitase Ultra were ln *v* = 10.11–3137.45/*T* (*R*^2^ = 0.990) and ln *v* = 15.68–4691.69/*T* (*R*^2^ = 0.991), respectively. The activation energy of Lecitase Ultra was higher than that of OM-PLA1 according to the absolute value of the equation slope. OM-PLA1 possessed higher catalysis efficiency than Lecitase Ultra. When the same temperature was increased, OM-PLA1 activity had lower increase rate than Lecitase Ultra.

### Effect of Metal Ions and Inhibitors on OMP-PLA1

**Figure 8:**
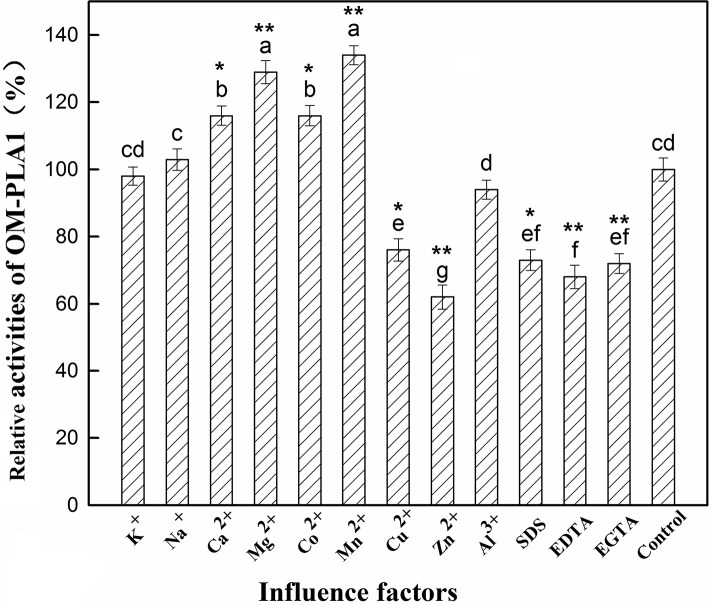
Effect of factors on outer membrane phospholipase A1 activity. The letters added on top of the bars were used to distinguish the significance between groups (*p* < 0.05). The presence of common letters between two groups indicated no significant difference (*p* > 0.05), whereas different letters between two groups indicated significant difference. * and **, respectively, indicated the test group had significant difference (*p* < 0.05) and extremely significant difference (*p* < 0.01) by comparing with control.

The effect of factors on OM-PLA1 activity was investigated under the conditions of 50°C and pH 7.5 (Figure 8). Ca^2+^, Mg^2+^, Co^2+^, and Mn^2+^ at 0.1 mM L^−1^ remarkably increased OM-PLA1 activity. Cu^2+^, Zn^2+^, SDS, EDTA, and EGTA at 1 mM L^−1^ considerably decreased OM-PLA1 activity. By contrast, 1 mM L^−1^ K^+^, Al^3+^, and 1 mM L^−1^ Na^+^ did not remarkably affect OM-PLA1 activity. The kinetic parameters of OM-PLA1 were determined by calculating the consumption amount by using the gradient concentrations of soybean lecithin as the catalytic substrate.

**Figure 9:**
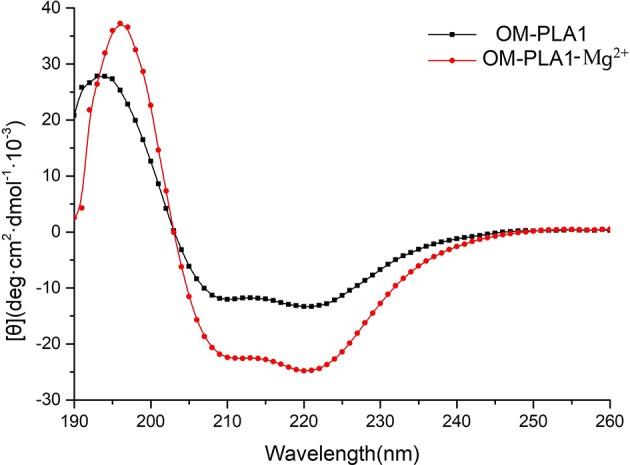
[θ] values of OM-PLA1 and OM-PLA1-Mg^2+^ under different wavelengths by circular dichroism spectroscopy.

**Figure 10:**
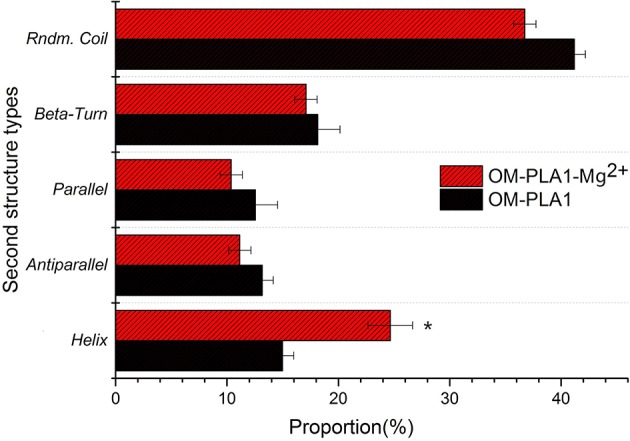
Secondary structures and proportions of OM-PLA1 and OM-PLA1-Mg^2+^ analyzed by CDPro software.

CD spectrum was used to determine the secondary structure of OM-PLA1 under the condition of 0.1 mM L^−1^ Mg^2+^ (Figure 9). The highest peak value [θ] of OM-PLA1 solution was 36.56 deg·cm^2^·dmol^−1^·10^−3^, which was 1.31-fold higher than that of the control (27.83 deg·cm^2^·dmol^−1^·10^−3^). The lowest valley value decreased to −24.82 deg·cm^2^·dmol^−1^·10^−3^ from −13.35 deg·cm^2^·dmol^−1^·10^−3^ at the wavelength of 220 nm. The structure types of OM-PLA1 indicated that 0.1 mM L^−1^ Mg^2+^ significantly increased helix proportion (Figure 10). The addition of Mg^2+^ decreased the proportion of other secondary structures (rndm.coil, β-turn, parallels, and antiparallel). The results indicated that the secondary structure change caused by metal ion Mg^2+^ increased the enzyme activity.

### Effect of Treatment Time on Crude Rapeseed Oil Degumming

**Figure 11:**
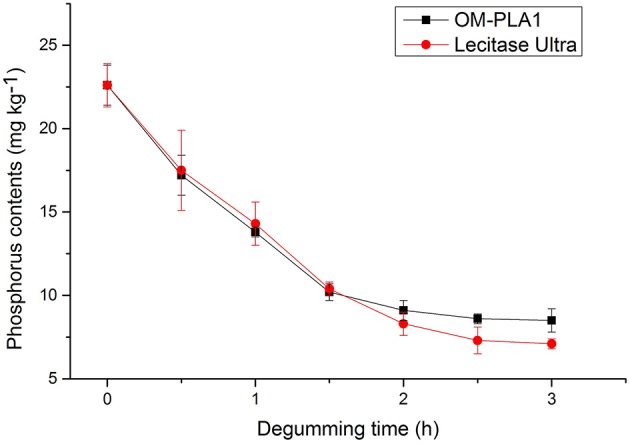
Effect of treatment time on the crude rapeseed oil degumming of OM-PLA1 and Lecitase Ultra.

Phosphorus contents below 10 mg kg^−1^ generally satisfied the requirement for edible oil. OM-PLA1 and Lecitase Ultra were used to degum crude rapeseed oil under the same conditions. The effect of treatment time on oil degumming was investigated by determining the phosphorus content (Figure 11). Catalysis was performed at 50°C with the addition of 15 U enzyme in 150 g of crude rapeseed oil containing 22.6 mg kg^−1^ phosphorus. After 2 h of processing, the phosphorus contents of OM-PLA1 and Lecitase Ultra decreased from 22.6 mg kg^−1^ to 9.1 and 8.3 mg kg^−1^. The final phosphorus content satisfied the edibility requirement of vegetable oils (i.e., <10 mg kg^−1^). At the initial catalytic stage, OM-PLA1 possessed almost the same degumming efficiency with Lecitase Ultra (<1.5 h). After treatment for 2 h, OM-PLA1 had lower residual activity than Lecitase Ultra. Therefore, the enzyme stability of OM-PLA1 was slightly lower than that of commercialized Lecitase Ultra during oil degumming.

### Amounts of OM-PLA1 and Lecitase Ultra Affecting Degumming

**Figure 12:**
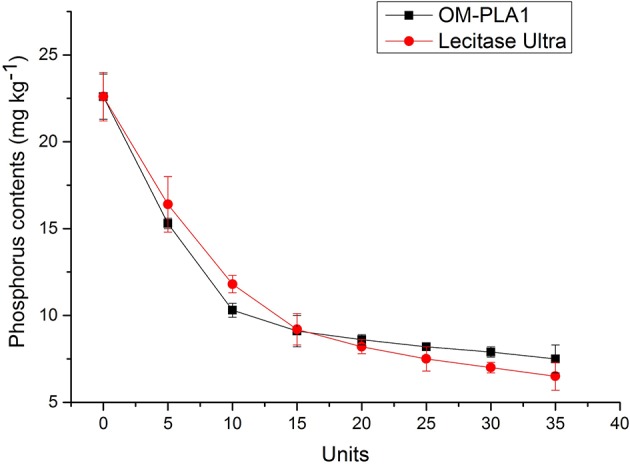
Effect of OM-PLA1 and Lecitase Ultra amount added on crude rapeseed oil degumming.

Different amounts of OM-PLA1 and Lecitase Ultra were added into 150 g of crude rapeseed oil adjusted to pH 7.5 containing 22.6 mg kg^−1^ phosphorus under the same conditions. Degumming was performed at 50°C for 2 h at a shaking speed of 150 rpm (Figure 12). The phosphorus content in 150 g of crude rapeseed oil decreased to 9.1 and 9.2 mg kg^−1^ with the addition of 15 units of OM-PLA1 and Lecitase Ultra, respectively. Appropriately 0.2 mL of OM-PLA1 broth (≥15 U) could meet the need for the enzymatic degumming of crude rapeseed oil. OM-PLA1 possessed lower catalysis efficiency than Lecitase Ultra when the amount of enzyme was >15 units. OM-PLA1 possessed almost the same catalysis efficiency compared with Lecitase Ultra. However, OM-PLA1 still had a large space to be modified to improve its catalytic performance.

## Discussion

Enzymatic characteristic is an important index reflecting the catalytic performance of enzymes. In the present study, the kinetic parameters of OM-PLA1 were compared with those of Lecitase Ultra. OM-PLA1 possessed higher substrate affinity and catalysis efficiency but lower Vmax and temperature stability than Lecitase Ultra. Lecitase Ultra is a chimeric enzyme that fuses with *Thermomyces lanuginosus* lipase and *Fusarium oxysporum* phospholipase A1. The stability of *T. lanuginosus* lipase is combined to the A1 phospholipase activity (Virgen-Ortiz et al., 2019). However, OM-PLA1 only exerts phospholipase activity without lipase activity, which can affect stability. Given its high catalytic efficiency, OM-PLA1 provides an alternative material to further modify this gene by molecular approach and produce excellent phospholipase for enzymatic degumming.

Metal ions bind to protein molecules by complexing with endogenous (side chain groups of amino acids) and exogenous ligands (such as H_2_O molecules, porphyrin rings, and organic small molecules) to form metal active sites (Liu and Xu, 2002). Metal sites exert significant effects on protein folding (Steif et al., 1995). Most helical structures affect enzyme stability and activity (Kang and Carey, 1999). In the present study, the secondary structure of OM-PLA1 contained several helixes, with the proportion of 14.98%. The helix proportion increased to 24.66% with the addition of 0.1 mM Mg^2+^. The Mg^2+^-binding region is a common core motif formed by folds and turn spirals. The orientation of the Mg^2+^-binding region is highly similar to the topological structure of the peptide chains (Kovall and Matthews, 1999).

In the present study, the expression of the *S. marcescens* phospholipase A1 gene, enzyme characterization, and crude rapeseed oil degumming OM-PLA1 were investigated. The result provided a reference for enzymatic degumming via a free enzyme approach. However, other areas still need to be improved, including (1) degumming effect of OM-PLA1 on other crude oil vegetable oils; (2) further exploring large-scale industrial applications to reduce the loss of enzyme activity; (3) combination use of OM-PLA1 and PLC for crude oil degumming; and (4) enzyme immobilization for recycling and reuse.

## Conclusion

The *S. marcescens* OM-PLA1 gene was expressed in engineered *E. coli* BL21(DE3). The highest activity of the recombinant OM-PLA1 with a size of appropriately 33 KDa reached 18.9 U mL^−1^. The optimum temperature and pH were 50°C and 7.5, respectively. The K_m_ values of OM-PLA1 and Lecitase Ultra were 18.53 and 42.1 mM, respectively. The Vmax values of OM-PLA1 and Lecitase Ultra were 1.35 and 1.72 mM min^−1^, respectively. The P content in 150 g of crude rapeseed oil decreased from 22.6 mg kg^−1^ to 9.3 mg kg^−1^ with the addition of 15 units of free OM-PLA1. OM-PLA1 possessed almost the same catalysis efficiency and lower enzymatic stability in comparison with Lecitase Ultra. The results provide an alternative phospholipase material for oil degumming.

## Data Availability Statement

The datasets generated for this study can be found in the Genbank sequence HG326223.1.

## Author Contributions

PY designed the experiment. YW performed experiment. SuJ performed characteristic determination. WX wrote the manuscript. ZZ analyzed the conclusions. ZH analyzed the data. DM measured the enzymatic activity. ShJ provided the research ideas. Y-HY searched the literature. All authors read and approved the final manuscript.

### Conflict of Interest

The authors declare that the research was conducted in the absence of any commercial or financial relationships that could be construed as a potential conflict of interest.

## Abbreviations

OM-PLA1: *Serratia marcescens* phospholipase A1 gene
EGTA, ethylene glycol-bis (β-aminoethyl ether)-N,N,N′: N′-tetraacetic acid
EDTA: ethylenediaminetetraacetic acid
PCR: polymerase chain reaction
SDS-PAGE: sodium dodecyl sulfate- polyacrylamide gel electrophoresis
IPTG: Isopropyl β-D-1-thiogalactopyranoside
AOCS: American Oil Chemists' Society.
